# Disease patterns of coronary heart disease and type 2 diabetes harbored distinct and shared genetic architecture

**DOI:** 10.1186/s12933-022-01715-1

**Published:** 2022-12-09

**Authors:** Han Xiao, Yujia Ma, Zechen Zhou, Xiaoyi Li, Kexin Ding, Yiqun Wu, Tao Wu, Dafang Chen

**Affiliations:** Department of Epidemiology and Biostatistics, School of Public Health, Peking University, Beijing, 100191 China

**Keywords:** Genetic architecture, Coronary heart disease, Type 2 diabetes, SNP-set approach

## Abstract

**Background:**

Coronary heart disease (CHD) and type 2 diabetes (T2D) are two complex diseases with complex interrelationships. However, the genetic architecture of the two diseases is often studied independently by the individual single-nucleotide polymorphism (SNP) approach. Here, we presented a genotypic-phenotypic framework for deciphering the genetic architecture underlying the disease patterns of CHD and T2D.

**Method:**

A data-driven SNP-set approach was performed in a genome-wide association study consisting of subpopulations with different disease patterns of CHD and T2D (comorbidity, CHD without T2D, T2D without CHD and all none). We applied nonsmooth nonnegative matrix factorization (nsNMF) clustering to generate SNP sets interacting the information of SNP and subject. Relationships between SNP sets and phenotype sets harboring different disease patterns were then assessed, and we further co-clustered the SNP sets into a genetic network to topologically elucidate the genetic architecture composed of SNP sets.

**Results:**

We identified 23 non-identical SNP sets with significant association with CHD or T2D (SNP-set based association test, P < 3.70 × ${10}^{-4}$). Among them, disease patterns involving CHD and T2D were related to distinct SNP sets (Hypergeometric test, P < 2.17 × ${10}^{-3}$). Accordingly, numerous genes (e.g., *KLKs, GRM8, SHANK2*) and pathways (e.g., fatty acid metabolism) were diversely implicated in different subtypes and related pathophysiological processes. Finally, we showed that the genetic architecture for disease patterns of CHD and T2D was composed of disjoint genetic networks (heterogeneity), with common genes contributing to it (pleiotropy).

**Conclusion:**

The SNP-set approach deciphered the complexity of both genotype and phenotype as well as their complex relationships. Different disease patterns of CHD and T2D share distinct genetic architectures, for which lipid metabolism related to fibrosis may be an atherogenic pathway that is specifically activated by diabetes. Our findings provide new insights for exploring new biological pathways.

**Supplementary Information:**

The online version contains supplementary material available at 10.1186/s12933-022-01715-1.

## Introduction

Coronary heart disease (CHD) and type 2 diabetes (T2D) are two complex diseases driven by numerous additive and interacting genetic factors and in combination with the environment. The two diseases represented the most prevalent and burdensome non-communicable chronic diseases (NCDs). Making the issue more challenging, the co-occurrence of CHD and T2D is also common rather than random assortment of individual conditions [1]. Diabetes mellitus confers an approximately two-fold increased risk of coronary heart disease (CHD), which in return serves as a major contributor to death and disability in T2D patients [2, 3]. Recent evidence also indicated that cardiovascular risk in T2D patients is highly heterogeneous [4]. Therefore, precise joint management of CHD and T2D for identifying patients with various risks for comorbidity is at high priority in clinical practice.

Genetic etiology for the complex interrelationships between CHD and T2D remains incompletely understood. Among the T2D patients, there has been shown a substantial genetic susceptibility for developing the subsequent cardiovascular outcomes [5, 6]. Previous analyses also identified that the locus on *GLUL,* which is functionally related to glutamic acid metabolism, was associated with elevated cardiovascular risk specifically in diabetic individuals [7, 8]. To clarify CHD patients with or without T2D, recent evidence further demonstrated a weak correlation of the genetic effects between CHD with T2D and CHD without T2D [9]. These initial observations indicated considerable distinctness in genetic architecture between different disease patterns involving CHD and T2D, hence requiring integrative discovery.

Genetic architecture refers to the number, frequency, and effect sizes of genetic risk alleles and their interactions with each other and the environment [10]. To understand genetic architecture, genome-wide association studies (GWAS) were conducted to determine the association between genomic DNA sequence variations and phenotypic variability, and have revolutionized the field of complex disease genetics over the past decade [10]. However, complex phenotypes present several challenges for the conventional analysis strategy based on additive models of individual variants, including the presence of epistasis, pleiotropy, heterogeneity, and involvement of multiple loci with small effects [11]. These factors have made it difficult to explain the cumulative functional effects of statistically associated loci and thus have limited the clinical predictive value of GWAS [12]. Accounting for the limitations, previous efforts have tried to group SNPs together for analyses over alternative tests of individual variants [11]. Major advantages of SNP-set analysis included replicability by alleviating the multiple testing burden, and the ability for handling complex disease by considering multiple variants in linkage disequilibrium (LD) and potential interactions between SNPs [13]. Recently, an unsupervised machine learning approach termed PGMRA was proposed by Zwir et al. for dissecting GWAS data into multiple SNP sets [14]. Of note, they demonstrated that genetic variants organized as clusters, acting in concert to influence heterogeneous traits [14–17]. On the other hand, Jorge et al. reported cumulative genetic effects associated with T2D, metabolic syndrome and obesity [18]. Their pioneering efforts provided adequate rationale for using the SNP set approach to specify complex interactive effects underlying the polygenic risk of complex disease.

GWAS studies have advanced considerable understanding of the genetic architecture individually for T2D and CHD, yielding the discovery of several dozen loci for each disease [19, 20]. However, past studies failed to consider the two diseases holistically, thus ignoring the genetic effects underlying multiple subtypes of CHD and T2D. Furthermore, the traditional analysis strategy based on individual SNPs limited the ability for capturing sufficient diversity of complex diseases distributed in subpopulations. Therefore, in the present study, we aimed to decipher the genetic architecture underlying multiple disease patterns involving CHD and T2D based on the SNP-set approach. Owing to the complex nature of both the phenotype and genotype, a genotypic-phenotypic architecture was raised for better decomposing their complex relationships (Additional file 1: Fig. S1). We used the unsupervised data-driven method to cluster SNP sets from CHD and T2D related variants and investigated their relations with different disease pattern subgroups of patients. We further topologically organized the interrelationships within SNP sets into genetic networks. It was postulated that the naturally joint relations between CHD and T2D were contributed by distinct but connected genetic architecture.

## Materials and methods

### Study participants

The study participants were included from the Fangshan Family-based Ischemic Stroke Study in China (FISSIC) [21]. FISSIC is an ongoing community-based case–control genetic epidemiological study that started in June 2005, which enrolls families in Fangshan District, a rural area located southwest of Beijing, China. A total of 1229 participants with available genomic data distributed across 513 families were recruited for the study. Our discovery sample proceeded with 441 unrelated participants randomly selected from each family, excluding 317 subjects with missing values for the diagnosis of CHD and T2D. The discovery sample consisted of 152 CHD and 158 T2D patients, including 61 subjects with CHD and T2D comorbidity, 91 subjects with CHD alone, and 97 subjects with T2D alone. The remaining 192 were control subjects with no CHD or T2D. We replicated the SNP set results in the remaining 471 subjects.

This study was approved by the Ethics Committee of the Peking University Health Science Center (Approval number: IRB00001052-13027), and written informed consent was provided by all participants.

### Data collection

In the FISSIC study, baseline data including sociodemographic status, education, occupation, diet, lifestyle, health behavior, and medical history, of all participants were collected through a face-to-face questionnaire survey by trained staff. The branchial-ankle pulse wave velocity (baPWV) values were tested with a BP-203 RPE III automatic arteriosclerosis detection device (Omron Health Medical Co., Ltd., China).The pulse wave in the brachial artery and the posterior tibial artery pulse were measured using an automated oscillo metric method. baPWV was then calculated by dividing the distance between two pulse wave measurement points by the time difference between two pulse waves. The larger the value, the higher the degree of arteriosclerosis. The detector automatically calculated and recorded the baPWV value, taking the average of the left and right baPWV as the baPWV value. For fasting blood glucose (fbg), after overnight fasting for at least 12 h, a venous blood sample was obtained from the forearm of each participant. Serum or plasma samples were separated within 30 min of collection and were stored at – 80 °C for measurement. Laboratory tests were performed by qualified technicians from the Laboratory of Molecular Epidemiology in the Department of Epidemiology at Peking University.

### Disease definition

The presence of T2D and CHD was confirmed by a qualified physician. In particular, the diagnosis of CHD was based on one or more of the following: (1) history of confirmed CHD, including myocardial infarction, angina pectoris, and ischemic cardiomyopathy; and (2) use of drugs for controlling CHD. The diagnosis of T2D was based on one or more of the following: (1) self-reported diabetes history; (2) hypoglycemic drug use; (3) fasting blood glucose (FBG) ≥ 7.0 mmol/L; and (4) two hours blood after glucose oral glucose tolerance test (OGTT) ≥ 11.1 mmol/L.

### Genotyping

DNA was extracted using a LabTurbo 496-Standard System (TAIGEN Bioscience Corporation, Taiwan, China). In addition, the purity and concentration of DNA were measured using ultraviolet spectrophotometry. Genomic DNA samples were genotyped on the Illumina Asian Screen Array. After prephasing using shapeit2, genotypes were imputed via IMPUTE2 from the 1000 Genomes Project phase 3, version 5 reference panel. Genotyped data underwent quality control using PLINK (v1.90b4.9 64-bit). Briefly, we excluded SNPs with missing rate ≥ 5% followed by the exclusion of SNPs with MAF ≤ 1%. We then removed SNPs with P-value < 1 $\times {10}^{-6}$ for Hardy–Weinberg Equilibrium. Samples with missing call rate ≥ 5% were excluded from the analysis.

### Statistical analysis

#### Identify SNP sets

Given genotype data from a GWAS represented as a matrix [SNPs $\times$ subjects], a SNP set is a submatrix comprised of a subgroup of subjects described by a particular subgroup of SNPs sharing distinct allele values [22]. To obtain comprehensive SNP sets with potential causal effects, we preselected SNPs for a loose association (*P* values < $5 \times {10}^{-5}$) with a global phenotype of CHD or T2D using the logistic regression model (Additional file 1: Fig. S2). We postulated that the multiple combination of CHD and T2D represented the integration of the two diseases, but not a new phenotype. Therefore, we pooled 110 variants associated with CHD and 83 variants associated with T2D with no overlap together as the initial genotypic database (with heritability of 43.2% and 38.2% respectively).

The nonsmooth nonnegative matrix factorization (nsNMF) method was conducted to enable an inference for SNP sets embedded in the SNP-Subject matrix (193 by 441) [23]. NMF decomposes the original matrix as a product of two matrices that are constrained by having nonnegative elements. Mathematically, this corresponds to finding an approximate factoring for ${{\varvec{X}}}_{m\times n}\sim {{\varvec{W}}}_{m\times k}\times {{\varvec{H}}}_{k\times n}$, where ***W*** is an m $\times$ k matrix that defines the decomposition model whose columns specify how much each of the subjects contributes to each of the k factors, and ***H*** is a k $\times$ n matrix whose entries represent the SNP allele values of the k factors for each of the n subject samples. By producing truly sparse components of the data structure, nsNMF achieves a satisfactory interpretability for the submatrices within different factors.

To uncover the genetic architecture composed of SNP sets from different domains of knowledge, we repeatedly applied nsNMF to generate multiple clustering results using various numbers of factor initializations (2 ≤ k ≤ $\sqrt{n}$, where n is the number of SNPs). This process can be interpreted as unsupervised biclustering, since we avoid any assumption about the ideal number of submatrices as well as prior knowledge of the subject’s clinical status (control or case). Once the factorization was done, the most representative features (SNPs) and observations (subjects) formed the SNP sets for each factor. It was performed by selecting the rows or columns with the highest values above a threshold, which was established as 60% of the highest value per row or column in the study. This selection process also contributes to fuzziness, where a subject or SNP can belong to multiple submatrices under each *k*. For each run of the basic factorization method (2 ≤ k ≤ $\sqrt{n}$), all SNP sets generated were named G_k_i, where 1 ≤ i ≤ k.

#### Description of the characteristics of SNP sets

A total of 135 possibly overlapping SNP sets were generated in the discovery samples. We considered three posterior indicators for describing the SNP sets: the risk for CHD and T2D (percentage of cases among all subjects within a SNP set), SNP composition (percentage of SNPs associated with CHD or T2D in GWAS), and the direction of the effect (percentage of SNPs with protective or risk effects). Of 135 SNP sets, only 5% and 4% showed a merge of SNP group or SNP effect direction, which suggested that SNPs with the similar properties tend to group together.

#### Perform SNP-set based association tests for SNP sets

Analyses for the association between each SNP set and disease phenotype was performed with the use of the SNP-Set Kernel Association Test (SKAT) [24]. This testing framework allows for complex SNP interactions and nonlinear effects and thus has the power for detecting their joint activity. The age, age squared, gender, BMI and ancestry (10 PCs) of the subjects were used as covariates. We filtered out sets of SNPs that did not show statistical significance after adjusting for all possible generated sets (135, from 2 to 16).

#### Discover latent genotype–phenotype architecture and genotypic network

Phenotype sets were encoded as subgroups harboring subjects described by different disease patterns between CHD and T2D. To uncover the genetic architecture underpinning different disease patterns, 4 subgroups characterized by different disease patterns were identified: subjects with comorbidity of CHD and T2D, with CHD alone, with T2D alone, and with none of them. Among them, the comorbidity set and CHD alone set can exactly include all CHD patients in the study (and the same for T2D).We co-clustered SNP sets with phenotype sets into relations using the Hypergeometric test on intersected subjects [25].

Since the SNP sets were recurrently generated from different levels of factors, there were numerous highly overlapped/redundant SNP sets. We employed the Jaccard coefficient (JC) to indicate the overlap of a pair of SNP sets in terms of SNPs or subjects. Two sets with overlapping SNPs or subjects over 0.8 (calculated by the Jaccard coefficient) were considered as redundant sets [26]. Optimization strategy was applied to select and assemble optimal, non-redundant SNP sets with the strongest association with phenotype sets using the P-value of Hypergeometric test as the measure of association strength [14]. After simplifying, we checked for significant relationships between SNP sets and phenotype sets based on the threshold using Bonferroni correction (*P* values < 2.17 $\times {10}^{-3}$). These relations characterize the genotypic-phenotypic architecture.

All reserved SNP sets were co-clustered by calculating the pairwise probability of intersected SNPs among them using the Hypergeometric statistics. This allowed us to characterize the relations among SNP sets and to identify SNP sets that were connected to each other by having certain SNPs in common, thereby composing genotypic networks.

#### Functional annotation and enrichment analysis

SNPs were mapped to likely affected genes using snpXplorer based on combined annotation dependent depletion (CADD) score, expression-quantitative-trait-loci (eQTL) and variant position [27]. All possible molecular consequences of each SNP in the function of the gene were considered in the analysis. The cardiometabolic phenotype influenced by the genes within each SNP set was annotated by GeneCards [28]. To elucidate potential functional differences between different disease patterns, gene ontology (GO) and Kyoto Encyclopedia of Genes and Genome (KEGG) enrichment analyses were performed using R’s clusterProfiler package [29]. Functions or pathways with significant enrichment were identified based on the criterion: adjusted P < 0.05.

## Result

### Identifying SNP sets as candidates for explaining the genetic etiology of CHD and T2D

**Fig. 1:**
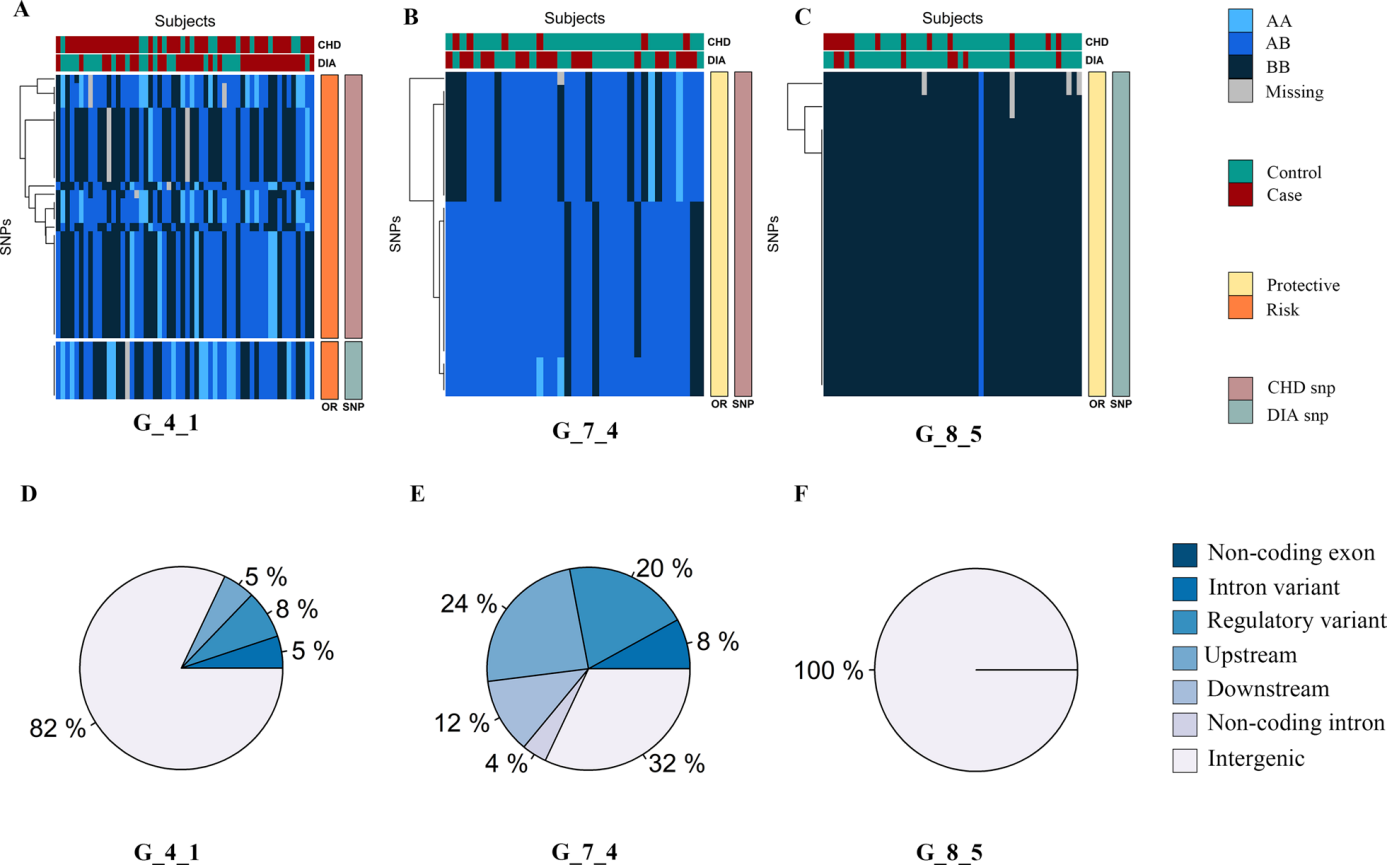
Examples of Identified Single-Nucleotide Polymorphism (SNP) Sets Represented as Heatmap Submatrices. Six examples of SNP sets are represented as heatmap biclusters (see supplemental figure S3 for all SNP sets). Allele values are indicated as BB (dark blue), AB (intermediate blue), AA (light blue), and missing (gray). Subject status (i.e., cases and controls) is annotated at the top of the heatmap: cases in red and controls in green. SNP composition (associated with CHD or T2D) and SNP effect direction (risk or protective) are indicated as colored bars at the right side. Genotypic SNP sets were labeled by a pair of numbers representing the maximum number of clusters and the order in which they were selected by the method with a prefix G for genotype. **A**–**C** Illustrate SNP sets with different combinations of SNP composition and SNP effect direction, which contributed to varied risk for CHD and T2D. The SNPs within each SNP set can map to different genomic positions and exhibit distinct molecular consequences. **D**–**F** present pie charts of the percentage of SNPs within each SNP set that belong to different types of consequence (see Additional file 1: Fig. S4 for molecular consequence in each SNP set)

We first applied nonsmooth nonnegative matrix factorization method recurrently to investigate SNP sets without prior biological knowledge. Our exhaustive search uncovered 23 nonidentical SNP sets, which varied in terms of allele value pattern and numbers of SNPs and subjects (Fig. 1; Additional file 1: Fig. S3). For example, G_7_4 contains 25 SNPs and 37 subjects, exhibiting a heterogeneous allele value pattern. Conversely, subjects in G_16_13 share relatively fewer SNPs (18 vs. 5), with all subjects holding the same interaction among a specific set of homozygotic alleles. Genome positions and molecular consequences of variants also appeared to be diverse within SNP sets, for the SNPs can map to multiple classes of genetic variants dispersed across all the chromosomes (Additional file 2: Table S1; Additional file 3: Table S2). Specifically, there were multiple SNPs within a SNP set annotated by different genes (e.g., G_2_1), multiple SNPs within a SNP set jointly affecting the same gene in different ways (e.g., rs7259003 and rs34227821 in G_7_4 with different consequences both mapped to *KLK5*), and different SNPs within different SNP sets mapped to the same gene (e.g., rs6134578 in G_10_7 and rs6078680 in G_4_1 both mapped to *SPTLC3*) (Additional file 6: Table S5).

For each SNP set, we continued to calculate the disease risk for CHD and T2D (percentage of cases in each set), SNP composition (percentage of SNPs associated with CHD or T2D in GWAS), and the direction of the effect (percentage of SNPs with protective or risk effects). There were 10 SNP sets comprised of SNPs associated with CHD, 7 comprised of SNPs associated with T2D and 6 SNP sets merged by SNPs from the two groups. Accordingly, 9 SNP sets were comprised of risk SNPs, 10 were comprised of protective SNPs and the remaining 4 SNP sets contained SNPs from both effect directions. Interestingly, it demonstrated a substantial interrelationship between the above three aspects within SNP sets, that is the risk SNPs tended to cluster with cases of corresponding disease while protective SNPs tended to cluster with controls (Fig. 1). As a result, variants with different properties integratively contributed to SNP sets characterized by heterogeneous disease patterns of CHD and T2D. For example, G_2_2 and G_4_1 were both composed of two groups of SNPs. However, G_4_1 with risk SNPs for both diseases yielded a higher proportion of subjects for CHD, T2D and their comorbidity, relative to G_2_2 with protective SNPs encoding mainly controls. In addition, one SNP set with risk loci for CHD can cohesively gather subjects of having CHD without T2D and subjects with comorbidity.

### SNP sets significantly associated with CHD and T2D

To capture the synergetic effect of multiple causal variants as well as possible epistatic effects within SNP sets, the association of SNP sets with coronary heart disease (CHD) and type 2 diabetes (T2D) was evaluated using the SNP-set Kernel Association Test (SKAT). All 23 SNP sets were significantly associated with CHD or T2D, reaching the significance threshold (3.7 × ${10}^{-4}$) set by Bonferroni correction for 135 generated sets (Table 1). Notably, SNP sets composed of variants for CHD and T2D also exhibited a significant association with either of the two diseases. 49 SNPs within G_2_1 and 7 variants within G_9_2 were initially associated with CHD in GWAS, yet also showed a significant association with T2D by the SNP-set based test. These SNPs can be mapped to 16 genes that are responsible for cardiometabolic traits. Similarly, 28 variants in 4 SNP sets mapped to 11 genes were found in relation to CHD through the SNP-set based test, with a loose association with T2D individually. Collectively, these results suggested the shared genetic architecture between CHD and T2D, in which some variants with small effects may jointly contribute to both CHD and T2D.

**Table 1:** Single-nucleotide polymorphism (SNP) sets reported significant association with coronary heart disease (CHD) or type 2 diabetes (T2D)

SNP set	SKAT *P* values		Subjects (N)	SNPs (N)	Disease risk (%)		SNP composition (%)		OR > 1 (%)
	CHD	T2D			CHD	T2D	CHD	T2D	
G21	1.52E−03	8.76E−05	159	81	48	48	60	40	74
G22	2.79E−07	1.18E−01	70	34	26	29	50	50	0
G41	3.44E−04	5.19E−01	56	39	75	61	82	18	100
G55	2.79E−07	1.18E−01	53	24	6	58	100	0	0
G63	3.42E−04	7.08E−01	43	14	63	44	100	0	100
G74	1.33E−07	7.23E−01	37	25	16	51	100	0	0
G82	2.69E−05	7.12E−01	128	10	52	45	100	0	100
G84	2.12E−05	1.77E−01	42	14	26	62	71	29	29
G85	1.00E+00	3.84E−05	50	14	26	18	0	100	0
G92	3.09E−01	9.19E−06	42	10	31	43	70	30	30
G107	3.04E−01	1.49E−05	45	8	58	62	0	100	100
G125	5.97E−01	3.16E−05	73	6	42	62	0	100	100
G162	2.12E−05	1.77E−01	34	10	29	35	100	0	0
G164	2.79E−07	1.18E−01	23	17	9	52	100	0	0
G167	2.69E−01	2.12E−05	50	17	42	20	0	100	0
G168	2.79E−07	7.18E−01	24	15	17	29	100	0	0
G169	2.42E−05	1.28E−01	66	4	41	55	50	50	50
G1610	1.39E−01	7.36E−06	39	4	33	5	0	100	0
G1611	2.91E−05	6.06E−01	88	9	42	42	100	0	100
G1613	1.00E+00	7.54E−05	18	5	39	67	0	100	100
G1614	2.12E−05	1.18E−01	57	4	65	44	100	0	100
G1615	1.28E−01	3.17E−05	19	7	63	53	0	100	100
G1616	2.60E−05	5.60E−01	72	7	21	33	100	0	0

Disease risk (CHD or T2D) was the percentage of cases within each SNP set. SNP composition was the percentage of SNPs associated with CHD or T2D in GWAS. OR > 1 represents the percentage of risk SNPs in each SNP set.

### Different disease patterns of CHD and T2D harbored distinct SNP sets, pathway enrichment and cardiometabolic trait levels

**Fig. 2:**
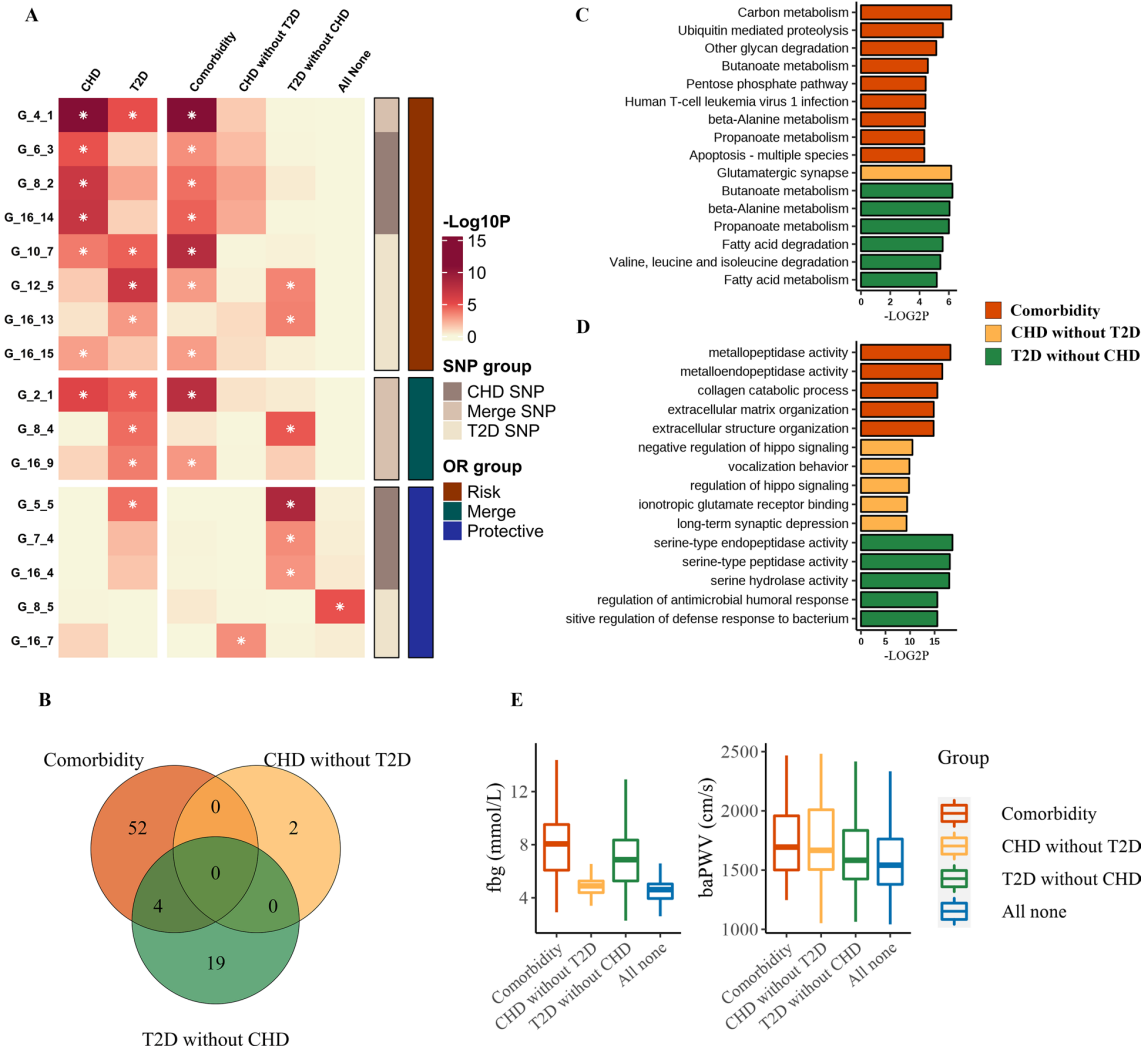
Different disease patterns with distinct genetic architecture and pathway enrichment. **A** Heatmap of associations of SNP sets with disease patterns of CHD and T2D. Hypergeometric analyses were performed based on common subjects between two sets. *P < 2.17 $\times {10}^{-3}$. The red bar indicates SNP sets composed of risk alleles, while the blue bar indicates SNP sets composed of protective alleles. Green bar corresponding to SNP sets containing variants for two effect directions. SNP sets for CHD variants are indicated in deep brown whereas SNP sets for T2D variants are indicated in light brown. **B** Venn plot showed the genes overlapping between different groups. **C** Significantly enriched KEGG pathways in different disease patterns. X-axis represents − ${log}_{2}$(*P*-value). The comorbidity group is indicated in red, the CHD without T2D group is in orange and the T2D without CHD group is in green. **D** The top 5 significantly enriched GO terms within each disease pattern. **E** Comparison of levels of cardiometabolic traits between disease patterns of CHD and T2D

Next, we examined whether the SNP sets were related to different disease patterns of CHD and T2D. By combining genotypic and phenotypic information, we uncovered a complex relationship between them: the same SNP set could be associated with multiple clinical outcomes (pleiotropy), while different SNP sets can relate to the same clinical outcome (heterogeneity). In addition, comorbidity groups were only encoded by SNP sets comprised of a majority of risk loci, while both protective and risk SNP sets connected to the CHD without T2D group or T2D without CHD group. Particularly, it demonstrated that genetic architecture was distinctly distributed in different subgroups for comorbidity, CHD alone, and T2D alone, except for only one SNP set (G_12_5) related to two disease patterns (Fig. 2). Furthermore, after annotation, there were only 4 common genes between comorbidity and T2D without CHD, with no overlap between the two groups and CHD without T2D (Fig. 2). This result may suggest that the three groups (CHD with T2D, CHD without T2D, T2D without CHD) were differentially related to distinct gene profiles.

To gain further support for the biological distinctness represented by SNP sets within different disease patterns, we assessed the variants in SNP sets for enrichment of functions and signaling pathways (Fig. 2; Additional file 4: Table S3; Additional file 5: Table S4). Globally, we found that disease patterns harboring distinct SNP sets were also differentially associated with various biological processes (*P* < 0.05). Within comorbidity sets, the most enriched KEGG pathway was carbon metabolism while the most enriched GO terms included metallopeptidase activity, collagen catabolic process and extracellular matrix organization. In comparison, the CHD without T2D group had distinct enrichment in glutamatergic synapse for KEGG pathway and hippo signaling, ionotropic glutamate receptor and neuron functions for GO terms. The T2D without CHD group was most strongly enriched in metabolic pathways, including butanoate metabolism, beta-alanine metabolism and fatty acid metabolism. The GO terms demonstrated a distinct enrichment in serine-type endopeptidase activity, serine-type peptidase activity and serine hydrolase activity. Interestingly, we also found a strong enrichment in immune functions within the T2D without CHD group. Collectively, we supposed that in each disease pattern, the SNP sets capture a different disease mechanism and thus localize largely to specific and distinct functions and pathways.

We also assessed whether levels of cardiometabolic traits were varied within different groups. Notably, fasting blood glucose (fbg) and branchial-ankle pulse wave velocity (baPWV) showed significant differences among comorbidity, T2D without CHD and CHD without T2D (*P* < 0.05, ANOVA). For instance, CHD patients with diabetes showed significantly higher levels of blood glucose, which may affect clinical treatment. Overall, our results addressed the importance of clarifying the presence of comorbidity in CHD and T2D patients.

### Relations among SNP sets mapped to disease patterns and to gene products

**Fig. 3:**
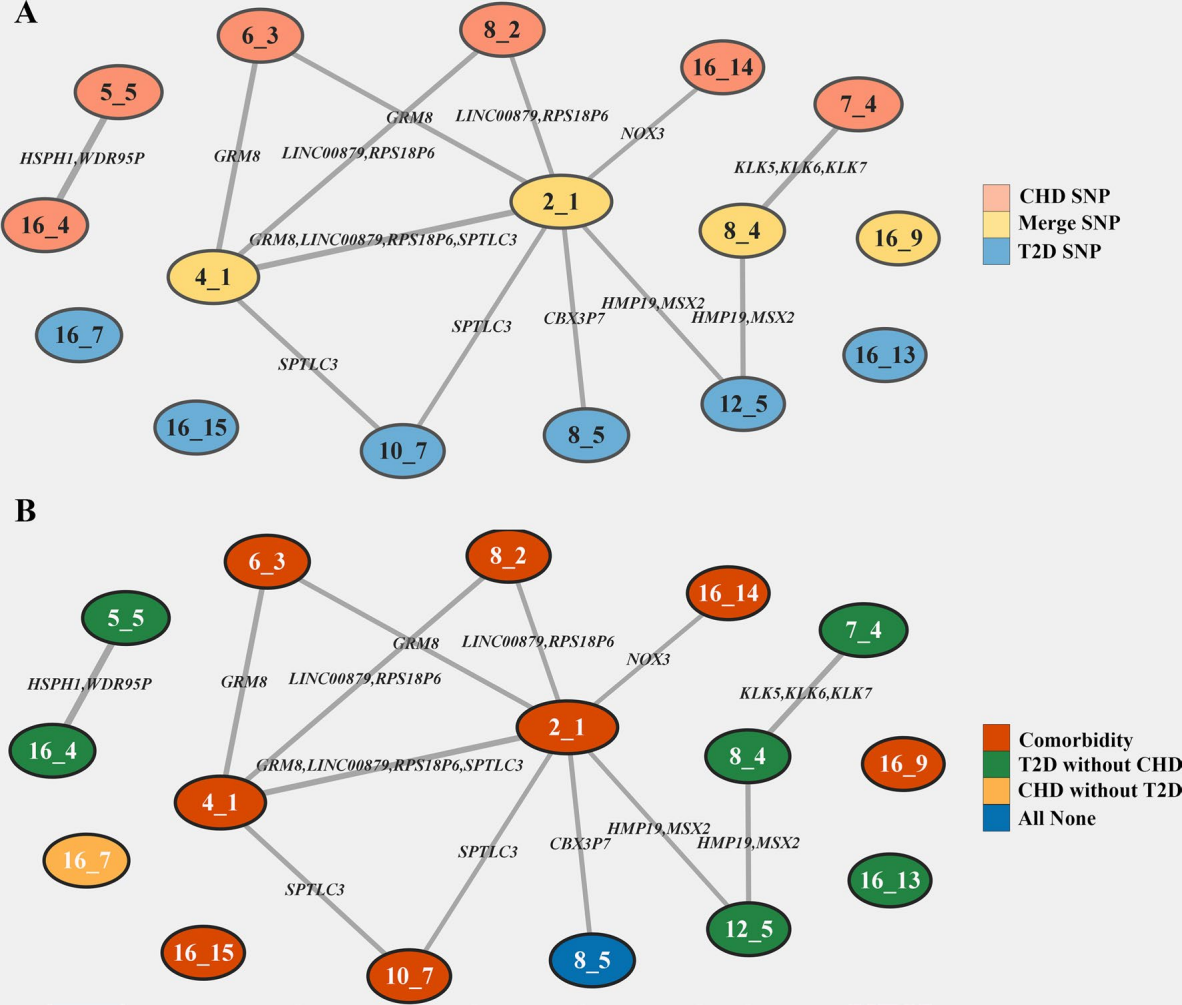
Genotypic networks for disease patterns of CHD and T2D. The genotypic network is depicted as nodes (SNP sets) linked by shared SNPs. 16 SNP sets significantly associated with phenotypic sets were topologically organized into 6 disjoint subnetworks, which suggested the heterogeneity in different disease subtypes. The edge width reflects the strength of overlap between two SNP sets computed by Jaccard’s coefficients. Shared genes enriched between two SNP sets are labeled on edges. The width of the node reflects the number of genes involved in each SNP set. **A** shows networks within SNP sets mapped to different SNP compositions. **B** presents SNP sets harboring different disease patterns. This network was visualized using Cytoscape 3.9.1

To intuitively establish the genetic architecture constructed by SNP sets, we interconnected the SNP sets into an organized network based on shared SNPs (Fig. 3). Generally, it demonstrated the heterogeneity, distinctness and connectivity of the genetic architecture of disease patterns involving CHD and T2D. For the heterogeneity, we found 6 disjoint sub-networks among 16 SNP sets, in which one highly connected network associated with 10 SNP sets, whereas four networks were composed of only a single isolated SNP set. In addition, within each disease pattern, the SNP-set networks were also disjoint.

Between different disease pattern groups, only two associations were identified, which additionally confirmed the distinctness mentioned above. Interestingly, there was a shared gene *CBX3P7* between the 2_1 set for comorbidity and the 8_5 set for subjects with no CHD or T2D. We inferred that this was because they involved the same SNPs but with different allele values (both alleles of a SNP can act as risk alleles in different genetic contexts) in different subjects [17].

In addition to sparseness, SNP sets within each disease pattern can co-cluster together with significantly overlapping variants. Linked pathways connected the SNP sets through shared gene products previously associated with cardiometabolic traits by GWAS. The emerging picture suggested that the disease patterns between CHD and T2D are a heterogeneous spectrum of diseases with some common genetic contribution in it.

### Replication of SNP sets in the remaining sample

Since our work was based on SNP sets, we evaluated the replicability of SNP sets in the remaining sample, which contained 87 subjects of comorbidity, 133 subjects with CHD without T2D, 93 subjects with T2D without CHD and 158 subjects having none of them. We evaluated the matching between SNP sets generated from the discovery sample and from the remaining sample using the same 193 variants. The probability of replication was measured by Hypergeometric test, with *P*-value = 0 considered as totally overlapped [26]. Remarkably, we found that 135 of 135 SNP sets in discovery sample were also generated with few differences in remaining sample. We suggested that the high replicability was due to the SNP sets holding similar allele value patterns in different populations.

## Discussion

In the present study, we performed an unsupervised, data-driven SNP set approach for uncovering the complex genotypic-phenotypic architecture of CHD and T2D disease patterns. We identified 23 non-identical SNP sets harboring variants with different genomic locations and molecular consequences, which also varied in composition and effect directions. Based on the SNP sets, key findings from our study include: (i) joint effects of multiple SNPs may explain the underlying genetic pleiotropy between CHD and T2D; (ii) subgroups of individuals with different disease patterns shared distinct genetic basis, which also affected different biological pathways; and (iii) genotypic network composed of SNP sets further showed the sparseness between different disease patterns with the connectivity within each subtype. Our work provides new insights into the genetic etiology of CHD and T2D.

In practice, the choice of the SNP-set formation strategy can influence the power of the approach [17]. Existing analyses grouped SNPs together into SNP sets based on a variety of genomic features such as physical location or biological functions [13, 30, 31]. However, it is reasonable to expect that we can extract the joint information at both the gene-level and pathway-level to improve the power for detecting true effects [24]. Here, we generated SNP sets by decomposing the GWAS data into multiple submatrices characterized by particular allele value pattern, and we suggested that this approach implied a more basic logic that captures the structure of both genotype and phenotype in a specific population. Correspondingly, we found a correlation between the SNP effect and the subject risk for T2D and CHD within each SNP set, which was ignored in Zwir’s study [14]. In addition, the SNP sets in our study can gather SNPs with different molecular consequences placed adjacently and mapped to the same gene. There were also SNP sets containing multiple genes that jointly enriched in the same functional pathway. Collectively, it was proposed that the data-driven clustering strategy for forming SNP sets is biologically interpretable.

## SNP set association detected shared genetic effects between CHD and T2D

GWAS studies have elucidated the shared genetic background and pathophysiology between coronary heart disease and type 2 diabetes [32]. A series of loci have been proven to be associated with both diseases, centering on atherosclerotic plaque destabilization [33], insulin regulation [34], and triglyceride metabolism [35]. In the present study, we detected plausible pleiotropic effects for CHD and T2D based on the SNP-set strategy. The SNP-set based association test allows for potential epistatic and nonlinear SNP effects, thus can substantially improve the power for detecting the true joint effects of multiple variants [36]. Along this line, we found that multiple variants associated with T2D individually may jointly influence CHD and vice versa, which suggested widespread pleiotropic effects contributed by interactive SNPs. Functional annotation further validated such pleiotropy. A total of 84 variants with plausible pleiotropic effects were assigned to 27 genes with a majority previously associated with cardiometabolic traits. For example, 6 pleiotropic loci within G_9_2 were annotated in *CABP1,* which was identified as risk gene for triglyceride levels [37]. We concluded that multiple variants with modest effects can cohesively affect a broad cardiometabolic basis for CHD and T2D. However, it can be argued that our results cannot distinguish biological pleiotropy and mediated pleiotropy, further validation by experimental study is encouraged [38].

## Distinct genetic architecture was related to comorbidity and CHD without T2D

Beyond the shared genetic basis between CHD and T2D, we additionally explored the genetic heterogeneity in different combinations of the two diseases. We uncovered the distinct but shared genetic architecture in four different disease patterns (comorbidity, CHD alone, T2D alone and all negative).Previous studies have tried to identify variants modulating the susceptibility to CHD specifically in diabetic patients. However, to date, the results have been mixed, as both differences and similarities have been found between CHD patients with or without diabetes [7, 8, 9, 39, 40]. Our findings provide evidences that the genetic architecture between the comorbidity group and CHD without T2D group is distinct rather than similar.

For the comorbidity of CHD and T2D, we discovered a heterogeneous genetic architecture composed of 4 disjoint SNP-set networks and the different genetic underpinnings further participated in multiple cardiometabolic mechanisms. For example, metabolic pathways enriched in KEGG analysis, such as the carbon metabolism [41], propanoate metabolism [42], beta-alanine metabolism [43], pentose phosphate pathway [44], butanoate metabolism [45], glycan degradation, and ubiquitin mediated proteolysis [46] all participate in the pathophysiology of CHD and T2D. Furthermore, we noted that the extracellular matrix, collagen catabolic, and metallopeptidase activity for GO term enrichment were unique to the comorbidity group, which also jointly contributed to the development and progression of fibrosis in diabetic cardiomyopathy [47, 48]. Specifically, imbalance of metallopeptidase in diabetic patients plays a key role in extracellular matrix modeling that favors fibrosis [48]. These results are noteworthy as they implicate cardiomyocyte fibrosis as a key pathological mechanism in the development of the co-occurrence of CHD and T2D, with distinct genetic basis contributing to an increased susceptibility subjected to comorbidity.

Compared to comorbidity, having CHD without T2D showed a globally different genetic architecture, gathering only one SNP-set with variants mapped to *SHANK2* and *SHANK2-AS1*. Genetic effects in this group appeared to be associated with the Hippo signaling pathway, which controls for lipid and glucose metabolism at both the cellular and organ levels [49]. It is also worth noting for the distinct enrichment in glutamate receptors and glutamatergic synapses. Evidence from experimental and human studies has pointed to glutamine/glutamic acid metabolism as contributing to the regulation of insulin secretion and glucose metabolism [50]. Remarkably, Qi et al. uncovered a diabetes-specific CHD loci functionally related to glutamic acid metabolism. A recent study may also support our results as they similarly discovered that the genetic effects linked with cardiac insulin resistance can lead to altered myocardial structure in non-diabetic individuals [51]. Since there was no genetic overlap between the CHD without T2D group and the comorbidity group, we suggested that the above effects were more sensitive to the CHD alone population. Furthermore, we speculated that the differentiation between comorbidity and CHD or T2D alone was conferred by two kinds of genetic effects: risk effects for comorbidity and protective effects for either of the diseases.

## Distinct and shared genetic architecture was related to comorbidity and T2D without CHD

There was distinct but also connected genetic architecture between comorbidity and T2D without CHD. For the distinctness, the genetic architecture of the T2D without CHD group comprised three disjoint SNP-set networks, with specific enrichment for fatty acid and branched-chain amino acids (BCAAs; isoleucine, leucine, and valine) metabolism. BCAA decomposition promotes fatty acid uptake and thus results in the accumulation of completely oxidized lipids and further the dyslipidemia [52]. Additionally, diabetic dyslipidemia and intra-myocardial lipid accumulation perform as key pathological features for diabetic cardiovascular disease [53]. GO enrichment in the T2D without CHD group was induced by profiles of *KLKs*, whose upregulation plays a distinct role in the pathogenesis of diabetic cardio endothelial damage and interacts with dysregulated lipid metabolism [54]. Collectively, our results may have clinical implications for preventing the development of CHD in T2D patients through targeting lipid acid metabolism.

For the similarities, firstly, there existed a SNP set characterized by mixed patients with comorbidity and T2D without CHD. Secondly, the two subtype groups shared 4 genes and thus common pathways such as butanoate metabolism and propanoate metabolism. In addition, variants mapped to *HMP19* and *MSX2* were significantly enriched between 8_4 set for T2D without CHD, 2_1 set for comorbidity, and 12_5 set mixed for the two phenotypes, in which both genes have been reported to be associated with lipid measurement [55, 56]. Potentially, the similarities of genetic effects between the two groups indicated that the pathophysiology of diabetes may be more critical for comorbidity, which is in part consistent with the fact that diabetes is a risk factor for coronary heart disease. Of note, as mentioned above, genetic effects for comorbidity were associated with fibrosis caused by diabetes. Given that fatty acid metabolism characterizing the T2D without CHD group is also responsible for fibrosis, we thereby suggested that the cardiovascular fibrosis occurring in diabetes patients is a potential therapeutic target for preventing the comorbidity of CHD and T2D.

## Strengths

The major strength of this study was that we concerned the complexity for both phenotype and genotype. For phenotype, we integrated CHD and T2D to stratify subpopulations with different disease patterns. For genotype, we performed a data-driven SNP-set approach to uncover the genetic architecture composed of multiple SNP sets with their interrelationships, accounting for joint effects of multiple variants. Combining the information of phenotype and genotype, our methods raised a genotypic-phenotypic architecture for better understanding the heterogeneity in multiple combinations of complex disease.

## Limitations

Several limitations should be acknowledged. Since SNP sets were generated by decomposing the GWAS data, the quality of the initial GWAS study was important for obtaining reliable results. Although we obtained robust replication for the SNP sets, a larger study would still be necessary for extending our results to a more general population. In addition, our findings are based on cross-sectional associations, so the comorbid diagnostic trajectories were not taken into account. Although the mean age of our subjects was over 55 years old, there may still exist subjects with CHD or T2D alone who will progress into comorbidity in the future, which may bias our results. Additionally, we only considered two chronic diseases as CHD and T2D. However, multimorbidity, defined as the coexistence of at least two chronic diseases in an individual, has become an increasing global public health concern. Collectively, a large-scale prospective study covering more disease patterns within multiple complex diseases is desired in subsequent studies.

Finally, though our framework can decipher the complex relationships between phenotypes and genotypes, a weakness of our data-driven approach was its lack of the power for effect size estimation and causal inference. Furthermore, since it was independent of any prior biological knowledge, the biological meaning of the findings relied on the following functional annotation and literature support. Therefore, while our results described the distinctness and similarity underlying genetic architecture encoding different disease patterns, and showed plausible biological meaning by functional annotation, additional fundamental work is still required before these associations can be thought as fully established.

## Conclusion

In summary, through a SNP-set approach, we demonstrated the distinctness and heterogeneity in the genetic architecture of different disease patterns involving CHD and T2D. Risk genetic effects for comorbidity and protective genetic effects subjected to CHD or T2D jointly contributed to this distinctness. In clinical practice, treating CHD and T2D separately is thereby inadequate. Lipid metabolism related to fibrosis may be an atherogenic pathway that is specifically activated by diabetes. Further studies are needed for validation.

## Supplementary Information

**Additional file 1: Figure S1.** Genotypic-phenotypic architecture for disease patterns of coronary heart disease (CHD) and Type 2 Diabetes (T2D). Genotype for CHD and T2D were intersected, composing of natural partitions of GWAS data (identified as sets of interacting single-nucleotide polymorphisms [SNPs] or SNP sets). Phenotype was identified as different disease patterns involving CHD and T2D, which occurred naturally in the general population. Genotypic-phenotypic architecture cross-matched the SNP sets network with phenotype subtypes. This schematic drew on previous work from Zwir et al. https://doi.org/10.1176/appi.ajp.2014.14040435. **Figure S2.** Manhattan plot summarizing the association results for the coronary heart disease (CHD) and Type 2 diabetes (T2D). Each tested SNP is visualised as a dot with location on the genome shown on the x-axis and -${log}_{10}$-transformed P values on the yaxis. Blue indicates SNP associated with CHD, red indicates SNP associated with T2D. Darkened colored dots above the dot-line indicated a loose genome-wide significance (P < 5x ${10}^{-5}$). **Figure S3.** Heatmaps of SNP sets. Abbreviations: CHD SNP: variant associated with CHD in logistic regression; T2D SNP: variant associated with T2D. **Figure S4.** Pie plots represents molecular consequence of SNPs within each SNP set.

**Additional file 2: Table S1.** Mapping SNP Variants to genomic information. (Information obtained from dbSNP database and GeneCards database).

**Additional file 3: Table S2.** Genes within each SNP set associated with multiple cardiometabolic trait.

**Additional file 4: Table S3.** KEGG enrichment among different disease patterns.

**Additional file 5: Table S4.** GO enrichment among different disease patterns.

**Additional file 6: Table S5**. Replication of SNP set using Hypergeometric test.

## Data Availability

The data presented in this study are available on request from the corresponding author. The data are not publicly available due to the policy of the Ethics Committee of the Peking University Health Science Center.

## Abbreviations

CHD: Coronary heart disease
T2D: Type 2 diabetes
SNP: Single-nucleotide polymorphisms
nsNMF: Non-smooth nonnegative matrix factorization
NCD: Non-communicable chronic disease
GWAS: Genome-wide association studies
LD: Linkage disequilibrium
SKAT: SNP-Set Kernel Association Test
JC: Jaccard coefficient
CADD: Combined annotation dependent depletion core
eQTL: Expression-quantitative-trait-loci
GO: Gene ontology
KEGG: Kyoto Encyclopedia of Genes and Genome
fbg: Fasting blood glucose
baPWV: Branchial-ankle pulse wave velocity
FISSIC: Fangshan Family-based Ischemic Stroke Study in China
