# Supplementary material for: Disease patterns of coronary heart disease and type 2 diabetes harbored distinct and shared genetic architecture
Source: Cardiovasc Diabetol. 2022 Dec 9;21:276. doi: 10.1186/s12933-022-01715-1 (PMC9738029; doi:10.1186/s12933-022-01715-1)
Supplement: Supplementary file 1 — Additional file 1: Figure S1. Genotypic-phenotypic architecture for disease patterns of coronary heart disease (CHD) and Type 2 Diabetes (T2D). Genotype for CHD and T2D were intersected, composing of natural partitions of GWAS data (identified as sets of interacting single-nucleotide polymorphisms [SNPs] or SNP sets). Phenotype was identified as different disease patterns involving CHD and T2D, which occurred naturally in the general population. Genotypic-phenotypic architecture cross-matched the SNP sets network with phenotype subtypes. This schematic drew on previous work from Zwir et al. https://doi.org/10.1176/appi.ajp.2014.14040435. Figure S2. Manhattan plot summarizing the association results for the coronary heart disease (CHD) and Type 2 diabetes (T2D). Each tested SNP is visualised as a dot with location on the genome shown on the x-axis and -${log}_{10}$log10-transformed P values on the yaxis. Blue indicates SNP associated with CHD, red indicates SNP associated with T2D. Darkened colored dots above the dot-line indicated a loose genome-wide significance (P < 5x ${10}^{-5}$10-5). Figure S3. Heatmaps of SNP sets. Abbreviations: CHD SNP: variant associated with CHD in logistic regression; T2D SNP: variant associated with T2D. Figure S4. Pie plots represents molecular consequence of SNPs within each SNP set. [file 12933_2022_1715_MOESM1_ESM.docx]

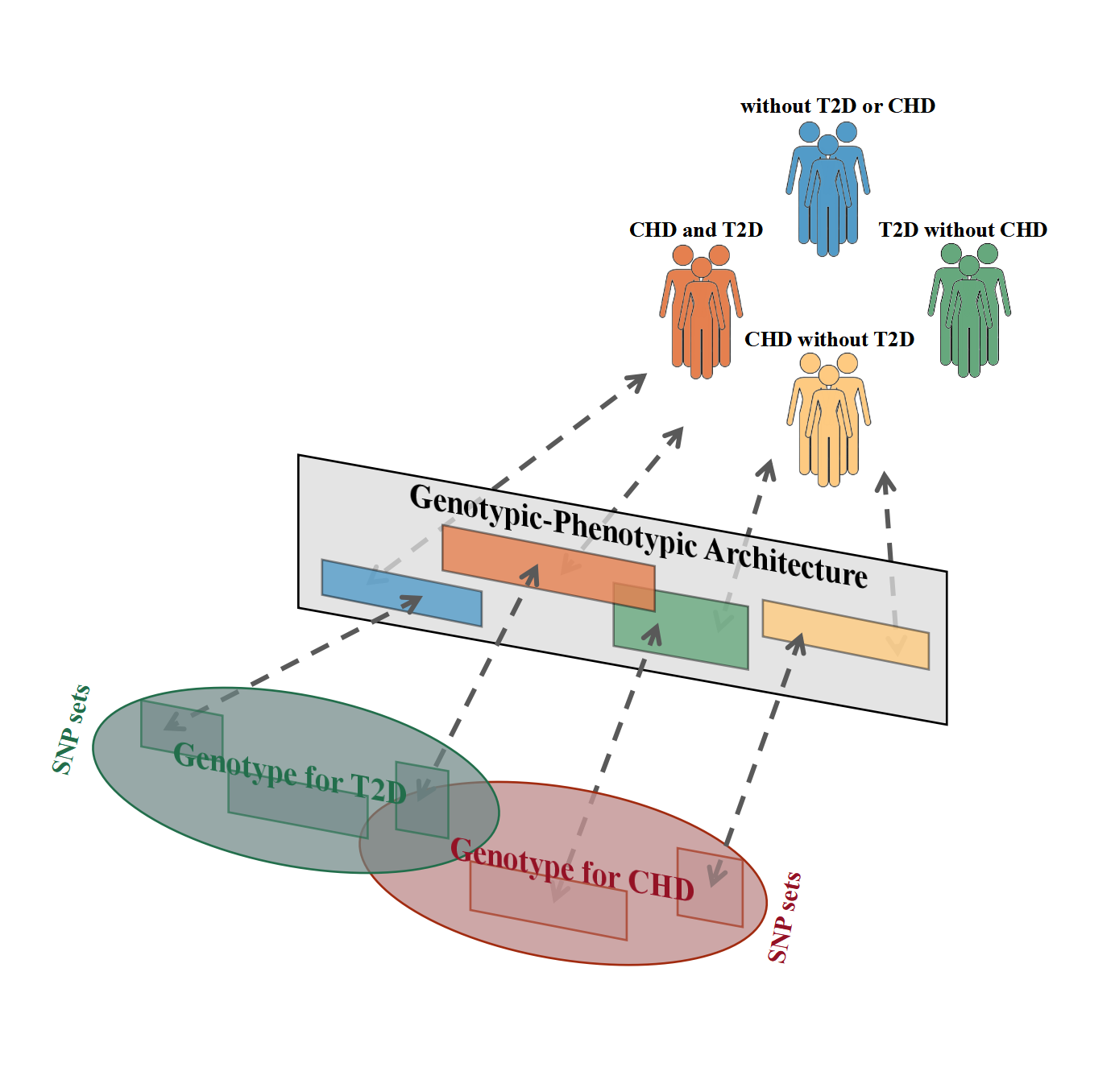


**Figure S1. Genotypic-phenotypic architecture for disease patterns of coronary heart disease (CHD) and Type 2 Diabetes (T2D).** Genotype for CHD and T2D were intersected, composing of natural partitions of GWAS data (identified as sets of interacting single-nucleotide polymorphisms [SNPs] or SNP sets). Phenotype was identified as different disease patterns involving CHD and T2D, which occurred naturally in the general population. Genotypic-phenotypic architecture cross-matched the SNP sets network with phenotype subtypes. This schematic drew on previous work from Zwir et al. doi: 10.1176/appi.ajp.2014.14040435


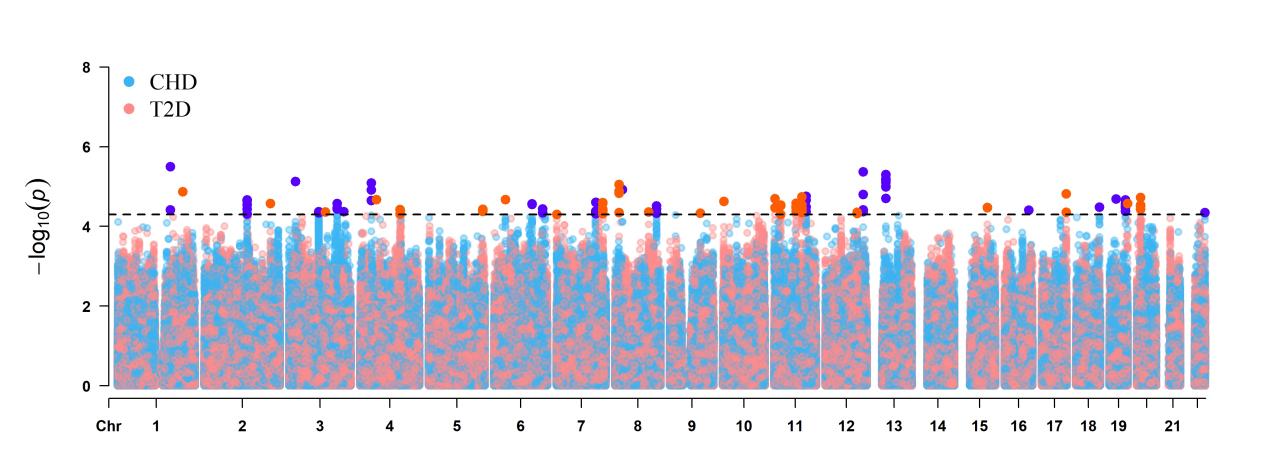


**Figure S2. Manhattan plot summarizing the association results for the coronary heart disease (CHD) and Type 2 diabetes (T2D).** Each tested SNP is visualised as a dot with location on the genome shown on the x-axis and -${log}_{10}$-transformed p values on the yaxis. Blue indicates SNP associated with CHD, red indicates SNP associated with T2D. Darkened colored dots above the dot-line indicated a loose genome-wide significance (p < 5x${10}^{-5}$).


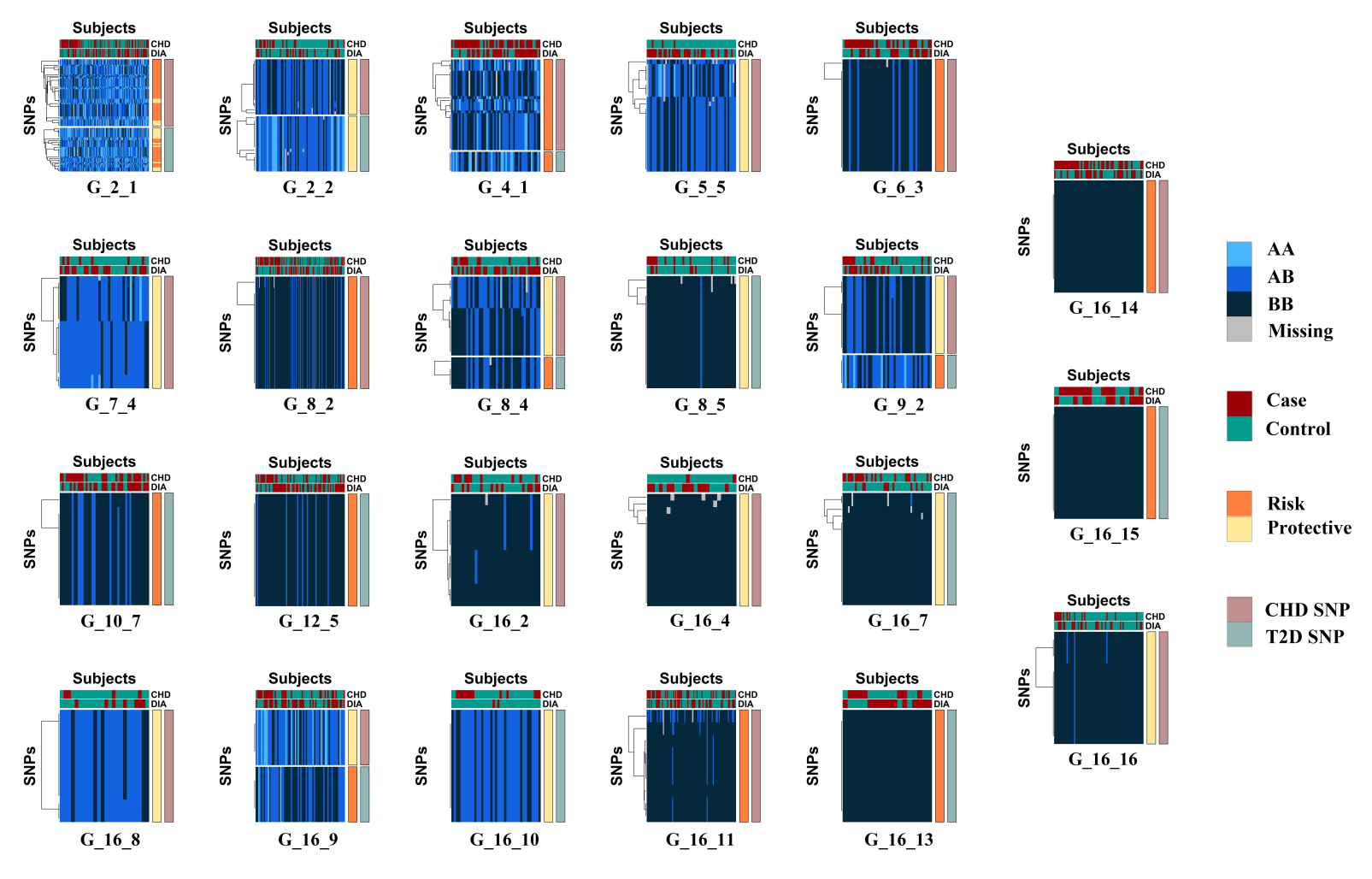


**Figure S3. Heatmaps of SNP sets.** Abbreviations: CHD SNP: variant associated with CHD in logistic regression; T2D SNP: variant associated with T2D

**
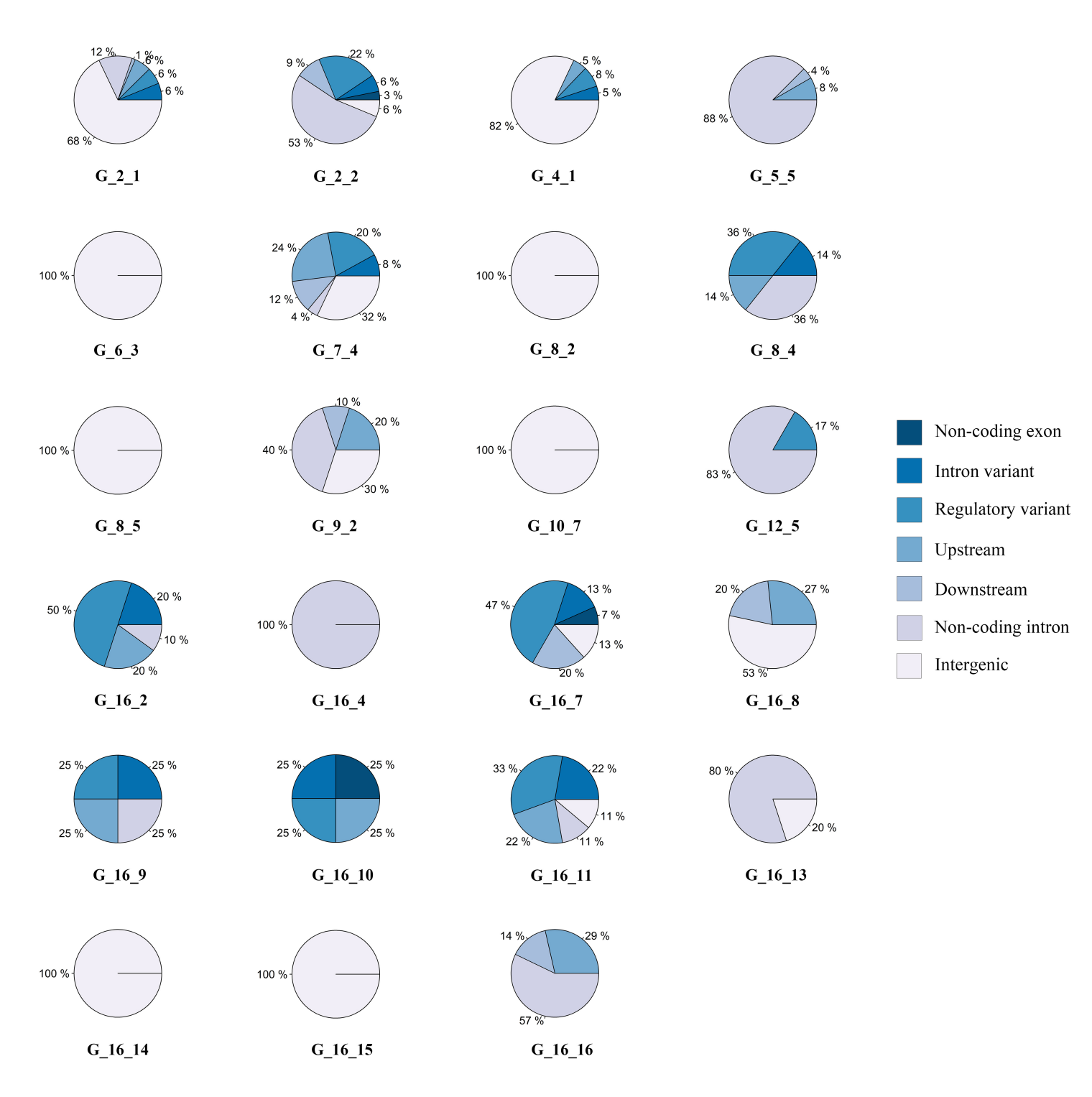
Figure S4. Pie plots represents molecular consequence of SNPs within each SNP set**
